# Clinical Evaluation of the Appropriateness Use Criteria for Single-Photon Emission-Computed Tomography: Differences by Patient Population, Physician Specialty, and Patient Outcomes

**DOI:** 10.5402/2011/798318

**Published:** 2011-06-02

**Authors:** Regina S. Druz, Lawrence M. Phillips, Gulru Sharifova

**Affiliations:** ^1^Department of Cardiology, North Shore University Hospital, Manhasset, New York, NY 11030-3816, USA; ^2^Division of Cardiology, New York University School of Medicine, New York, NY 10016, USA; ^3^Feinstein Institute of Biomedical Research, Graduate Clinical Research Center, Manhasset, New York, NY 11030, USA

## Abstract

*Objectives*. Determine outcome of the 2005 appropriateness use criteria (AUC) for SPECT in a diverse population of patients and physicians. *Background*. AUC for SPECT were the first cardiology document to identify 52 clinical indications for imaging, 49 of them for stress SPECT. AUC have been proposed as cornerstone of responsible use of perfusion imaging. *Methods*. 585 consecutive patients undergoing SPECT were evaluated prospectively. Appropriateness was examined for demographic variables, clinical variables, and for physician and patient subgroups. Combined end-point of total mortality, cardiac revascularization, and cardiac admissions at 1 year post SPECT was evaluated. *Results*. SPECT indications were: appropriate, 63%; uncertain, 20%; inappropriate, 14%; not assigned, 3%. Most appropriate SPECT were observed in patients with known coronary disease (72%), chest pain syndrome (89%), high pre-test likelihood of disease (100%), men (70%), inpatients (72%), and cardiovascular physicians' referrals (69%). End-point was reached in 53 patients (97.4% follow up). Unadjusted event rates were: appropriate (12%), uncertain (7.1%), inappropriate (2.4%) SPECT (*P* = .01). *Conclusion*. Appropriateness of SPECT differs in subgroups of patients and physicians. Clinically significant outcomes occur more frequently in the appropriate stress SPECT group. Focused efforts are need for outpatients, asymptomatic patients, women, and non-cardiovascular physicians.

## 1. Introduction

There has been an explosive growth in cardiovascular imaging with stress testing demonstrating 6.1% annual increase versus 2% for cardiac catheterization, 0.8% for percutaneous intervention, and 0.1% for acute myocardial infarctions in population-based study of the United States Medicare patients from 1993 to 2001 [1]. Increased use of cardiac imaging has been under scrutiny with the Center for Medicare and Medicaid Services, and significant cuts to SPECT payment schedules were implemented under the Deficit Reduction Act in November 2009. Additionally, insurance payers contracted with radiation services benefit managers to oversee utilization of cardiac imaging further limiting access to imaging. 

In response to these developments, the American College of Cardiology pioneered appropriateness criteria for single-photon emission computed tomography (SPECT) myocardial perfusion imaging (MPI) in 2005. The criteria were developed to account for evidence-based clinical relevance of stress perfusion imaging and were the first cardiology-specific document to address appropriateness. The criteria relied on the modified RAND/UCLA methodology to identify 52 common clinical scenarios that were divided by level of appropriateness into appropriate, uncertain, and inappropriate indications [2, 3]. 49 indications addressed stress SPECT, 1 viability, and 2 radiocineangiography. Appropriateness criteria were subsequently developed for stress echocardiography, cardiac computed tomography, cardiac magnetic resonance imaging, and coronary revascularization [4–6]. SPECT criteria were reviewed in 2007 with new designations suggested for some indications [7]. An update on appropriateness criteria was published in May 2009 [8]. Appropriateness criteria became incorporated as a mandatory requirement by the Intersocietal Commission of Accreditation of Nuclear Laboratories starting in 2010. The American Recovery and Reinvestment Act of 2009 has endorsed a concept of comparative effectiveness that needs to be applied to cardiac imaging, and appropriateness criteria are expected to play a central role in that process [9]. In September of 2010, Center for Medicaid and Medicare Services has solicited an evaluation of point-of-order and point-of service determination of appropriateness in Medicare beneficiaries across a broad range of physicians and settings: SPECT MPI was the only cardiac procedure included. (http://www.cms.gov/DemoProjectsEvalRpts/downloads/Medicare_Imaging_Demonstration.pdf). To encourage the best use of AUC and decrease inappropriate imaging, the ACC national quality initiative Imaging in “Focus” (http://www.cardiosource.org/Science-And-Quality/Quality-Programs/Imaging-in-FOCUS.aspx) contains multiple resources pertaining to the AUC, including a decision algorithm has been developed by the ACC in partnership with Skyscape [10]. A free smartphone AUC application based on 2009 guidelines has been released (http://www.astellasapps.com/).

Despite these developments, the clinical use of the appropriateness criteria has not become standard of practice for physicians. Gibbons et al. have investigated performance of appropriateness criteria for stress SPECT and stress echocardiography in 284 and 298 patients, respectively [11]. Overall, 64% of stress studies were appropriate. 14% of SPECT and 18% of stress echocardiography studies were performed for inappropriate indications, and approximately 10% of all patients were unclassifiable. In a prospective multicenter trial, the appropriateness criteria were evaluated in 7 physician practices of various size in partnership with United HealthCare. Overall, 14% of all studies were inappropriate, most in asymptomatic individuals [12]. 

The objectives of the current study were to identify clinical value of 2005 AUC in various patient and physician groups and to focus on downstream use of resources as defined by patient outcomes in relation to appropriateness. We have prospectively applied appropriateness criteria to a diverse population of patients and physicians to evaluate:

1. impact of demographic (gender) and clinical (known coronary disease, symptoms, pretest likelihood, Framingham risk scores (FRS), and admission status) factors,
2. impact of referring physician specialty and practice type,
3. outcomes at 1 year after SPECT (combined end-point of total mortality, coronary revascularization, or admission for cardiac reason other than revascularization).

## 2. Methods

### 2.1. Patient Population

Over 3,000 patients undergo SPECT at our institution annually. From March 2007 thorough April 2008, consecutive patients older than 18 yrs of age were enrolled if they were English speaking, had a capacity for primary decision making, had no chronic illnesses or malignancy with estimated life expectancy of less than 1 year, and were willing to provide informed consent for access to their medical record, phone call followup and/or social security number. The final cohort consisted of 585 patients. 

Admission status was ascertained at the time of SPECT as inpatient or outpatient based on registration record. Reason for stress, type of stress, known cardiac history (defined as prior myocardial infarction or revascularization), risk factor profile, and name of the referring physician were obtained and recorded. Followup was complete in 554 patients (94.7%).

### 2.2. Physician Specialty Verification

Referring physician name for each patient was cross-referenced with the hospital roster of credentialed physicians for both voluntary and full-time faculty. All cardiology and cardiac surgery physicians were designated as cardiovascular (CV MD). All other physicians were designated as noncardiovascular (non-CV MD). There were 258 patients referred by CV MDs, and 327 referred by non-CV MDs.

### 2.3. Appropriateness Criteria

Appropriateness criteria tables were included in each patient's file upon recruitment into the study [3, 13]. Appropriateness was determined prospectively based on symptoms, pre-test likelihood of disease [14], Framingham risk scores, FRS [15], and presence or absence of 49 stress SPECT MPI indications as identified during patient interview. While pre-test likelihood of disease was determined for all patients, FRS were estimated for asymptomatic patients without known coronary artery disease. The following assumptions were made.

1. Lipid values were often not available for calculation of FRS. Total cholesterol and HDL cholesterol were assigned 0 points if no dyslipidemia or treatment with statins were reported, and 1 point if reported.
2. Any chest pain syndrome or anginal equivalent were recognized as symptoms.
3. If more than one indication was identified, the most appropriate for SPECT was chosen by consensus opinion of investigators.

### 2.4. Statistical Analysis

Chi-square was used, and Fisher exact test was substituted for chi-square as indicated by frequencies of observations. SAS 9.1 (Cary, NC) was used for analysis.

## 3. Results

### 3.1. Patient Population

Patient characteristics are presented in Table 1. Overall, 570 (97%) of patients were assigned by appropriateness indications. Among 15 (3%) patients not classifiable 3 were inpatients, 12 were outpatients, 12 were men, 3 were women, 8 had known CAD, and 7 had no CAD. 7 unassigned patients were asymptomatic: 2 with a low FRS, and 5 with an intermediate FRS.

Two site-specific patterns of care are important to mention. Our emergency room policy expedites admission to the in-house hospitalist service for any patient with a “soft rule out MI”, thus many of them were inpatients at the time of SPECT MPI. Additionally, despite a rather high number of patients with symptoms (53%), only 6% of patients were in the high pre-test category reflecting a bias toward invasive coronary angiography.

### 3.2. Indications for SPECT

**Figure 1:**
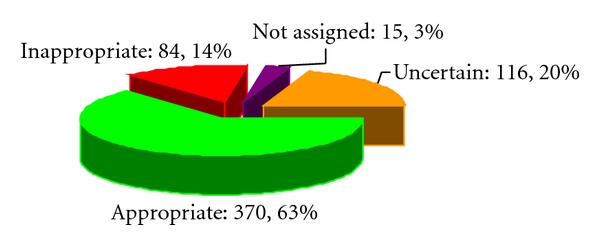
Distribution of studies in each of the appropriateness categories. Appropriate (green), uncertain (gold), inappropriate (red), not assigned (purple). Numbers of patients in each category and percent of all patients (rounded to the nearest whole value) are shown next to the corresponding symbols.

570 (97%) patients were classified by clinical indications with 15 (3%) not assigned (Figure 1). Most commonly observed indications in our patient population are listed below by category (% of all patients). Additional indications were observed in ≤1% of the patients.

#### 3.2.1. Appropriate Indications

Of all stress SPECT studies, 63% were *appropriate* with the most common indications as follows:

1. detection of CAD, symptomatic: 37%
  - intermediate pre-test probability, ECG interpretable and able to exercise: 21%; ECG uninterpretable or unable to exercise: 12%,
  - high pre-test probability, ECG interpretable and able to exercise: 3%; ECG uninterpretable or unable to exercise: 2%,
2. Risk assessment: 23%
  - after revascularization (PCI or CABG): 12%
    - symptomatic, evaluation of chest pain: 7%,
    - asymptomatic prior to CABG, ≥ 5 yrs after CABG: 3%; symptomatic prior to CABG, ≥5 yrs, 3%,
  - preoperative evaluation for noncardiac surgery intermediate risk predictor or poor exercise tolerance (<4 METS): 4%,
  - with prior test results: 4%
    - asymptomatic or stable symptoms, abnormal catheterization or prior SPECT ≥2 yrs to evaluate worsening disease: 2%,
  - high FRS, asymptomatic: 3%,

#### 3.2.2. Uncertain Indications

*Uncertain* indications for SPECT were observed in 20%. The most common uncertain indications were.

1. detection of CAD: asymptomatic, moderate FRS: 15%,
2. risk assessment: 5%
  - after revascularization, symptomatic prior to revascularization ≥2 yrs after PCI: 4%; asymptomatic, ≥2 yrs after PCI: 1%.

#### 3.2.3. Inappropriate Indications

Inappropriate indications were observed in 14%

1. detection of CAD: 13%
  - asymptomatic, low FRS: 7%,
  - symptomatic, ECG interpretable and able to exercise: 6%,
2. risk assessment: 1%
  - Preoperative evaluation prior to non-cardiac surgery, minor to intermediate risk predictor, normal exercise tolerance (>4 METS): 1%.

There were 15 (3%) unclassifiable patients. The observed indications were: syncope [1], intermediate Duke treadmill score, low FRS [1], new onset atrial fibrillation, moderate FRS [2], asymptomatic, prior myocardial infarct of unknown age [2], symptoms unknown before prior PCI less than 2 yrs [3], known CAD, failed PCI [2], normal myocardial perfusion imaging more than 2 yrs ago, and moderate FRS [4]. 

While most of the appropriate, uncertain, and inappropriate indications observed in our patients were similar for 2005 and 2009 AUC, most of the unassigned patients were categorized as uncertain by 2009 AUC as shown in Table 2.

#### 3.2.4. Appropriateness of SPECT by Symptoms and Pretest Likelihood

**Figure 2:**
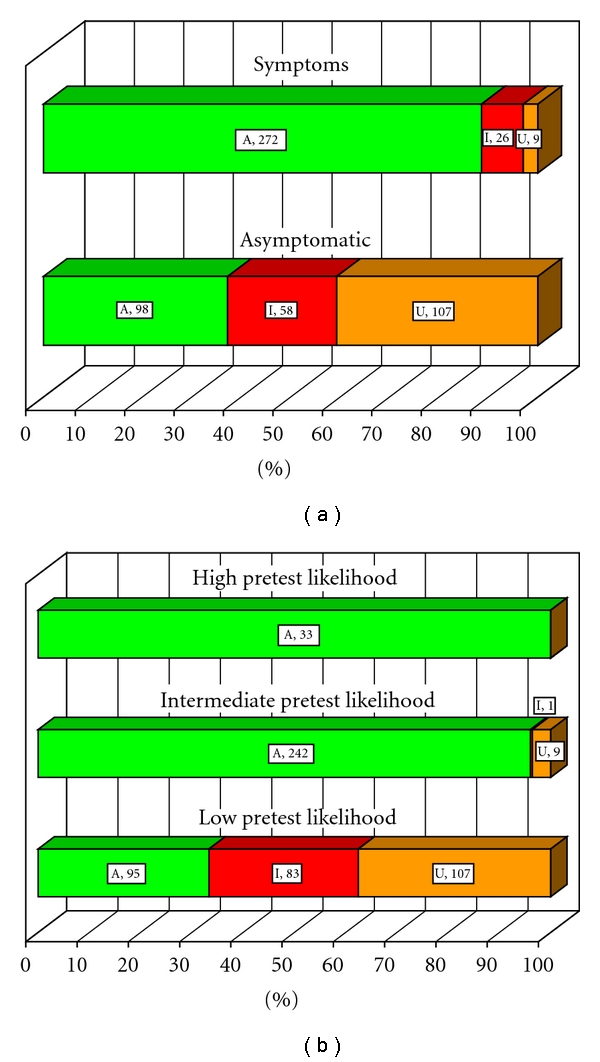
Appropriateness of SPECT by (a) symptoms and (b) pretest likelihood of disease ((a), (b): *P* < .0001). Symptoms refer to chest pain or anginal equivalent. Pretest likelihood: low, intermediate, or high based on age, gender, and symptoms. A, appropriate (green); U, uncertain (gold); I, inappropriate (red). Number of patients is shown next to the corresponding symbols for each of the appropriateness designations. Percent within each category is reflected on the horizontal axis.

As expected**, **symptoms (Figure 2(a)) and pre-test likelihood of disease (Figure 2(b)) significantly affected SPECT appropriateness. Appropriate studies were observed in 88% of patients with symptoms, in 96% of an intermediate pre-test likelihood group, and in 100% of a high pre-test likelihood group. Most of the inappropriate SPECT were observed in asymptomatic and low pre-test likelihood patients (58/84 and 83/84, resp.). Most of the uncertain stress SPECT were also observed in asymptomatic and low likelihood patients (106/116 and 107/116, resp.). Pretest likelihood did not differ by gender or known CAD.

### 3.3. Appropriateness of SPECT in Asymptomatic Patients

**Figure 3:**
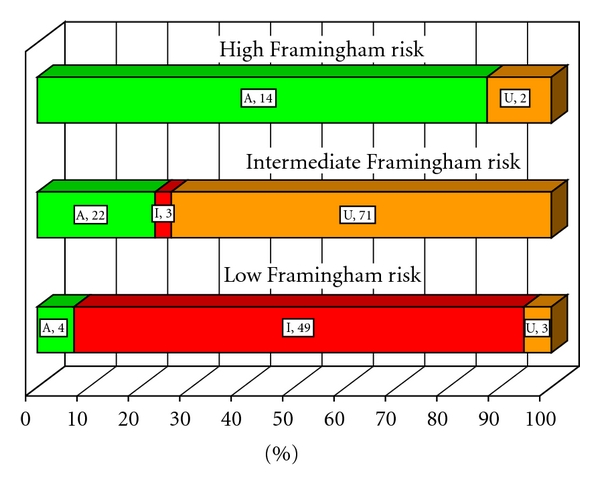
Appropriateness of SPECT in asymptomatic patients without known CAD stratified by Framingham risk scores (*P* < .0001). A, appropriate (green); U, uncertain (gold); I, inappropriate (red). Number of patients is shown next to the corresponding symbols for each of the appropriateness designations. Percent within each category of Framingham risk is reflected on the horizontal axis.

Asymptomatic patients without CAD were analyzed for stress SPECT appropriateness using FRS (Figure 3). Of the 175 patients, 7 were not assigned by appropriateness criteria. In the remaining group of 168 patients, FRS were low in 56 (33%), intermediate in 96 (57%), and high in 16 (9%). Based on age and gender, 160 had low pre-test probability of disease, and 8 were in the intermediate probability group. Most high FRS patients had appropriate SPECT (88%), those with an intermediate FRS had mostly uncertain SPECT (74%), while patients with low FRS had mostly inappropriate SPECT (88%).

### 3.4. Appropriateness by Admission Status, Gender, and Known CAD

Admission status, gender, and known CAD influenced SPECT appropriateness (Table 3).

#### 3.4.1. Admission Status

In comparison to outpatients, inpatients were more symptomatic (65% versus 43%, *P* < .0001), and at a higher pre-test likelihood risk (low: 44% versus 57%, intermediate/high: 56% versus 43%, *P* = .02) but did not differ by gender, known CAD, or FRS. Most inpatients (66%) were referred by non-CV MDs (Table 2). Most inpatients had appropriate stress SPECT studies (72%) with inappropriate SPECT similar to outpatients (15% and 14%, resp.). More uncertain SPECT (27% versus 13%) was observed in outpatients.

#### 3.4.2. Gender

Women had more symptoms (57% versus 49%, *P* = .04), and less known CAD (15% versus 39%, *P* < .0001) but did not differ in pre-test likelihood categories as compared with men. FRS were different for asymptomatic women as compared to men with more women in the low, and fewer women in the high risk groups: low, 41% versus 24; intermediate, 54% versus 61%; high, and 5% versus 15%, *P* = .01. Most women were referred by non-CV MDs (66%) with more even distribution among specialty of physicians for men (Table 2). As compared to men, women had less of appropriate SPECT (59% versus 70%) with nearly a three-fold difference in inappropriate SPECT (22% versus 9%).

#### 3.4.3. Known CAD

Compared to patients without known CAD, patients with CAD had fewer symptoms (42% versus 58%, *P* = .0007). More men (39%) than women (15%) had known CAD (*P* < .0001). 72% of all known CAD patients were referred by CV MDs. Patients with known CAD had more appropriate SPECT (74% versus 61%), and the least of inappropriate SPECT (4% versus 19%, *P* < .0001).

### 3.5. Appropriateness by Physician Specialty

There were differences in admission status, gender, and known CAD by referring physician specialty (Table 4). Most inpatients (66%) were referred by non-CV MDs. As expected, the majority of patients with known CAD were referred by CV MDs (72%). More men than women were evaluated by CV MDs. CV MDs outperformed non-CV MDs (Table 5) with more appropriate, and less inappropriate SPECT referred (69% versus 62%, and 10% versus 18%, resp., *P* = .03). Among CV MDs, private practitioners had a trend for more appropriate SPECT than full-time faculty cardiologists (74% versus 63%) although this difference was not significant. As described above, more women and inpatients were referred by non-CV MDs while more men, outpatients, and patients with known CAD were referred by CV MDs.

### 3.6. Outcome Analysis by Appropriateness Category

Follow up was completed in 554 patients: 359 with appropriate, 113 with uncertain, and 82 with inappropriate SPECT. Combined end-point of total mortality, revascularization and hospital admission for a cardiac reason other than revascularization was reached in 53 patients. There were 20 death (17 in appropriate SPECT patients), 14 revascularizations (13 in appropriate SPECT patients), and 28 admissions (21 in appropriate SPECT patients). Unadjusted events rates were 12% (43 of 359 patients) for appropriate, 7.1% (8 of 113 patients) for uncertain, and 2.4% (2 out of 82 patients) for inappropriate SPECT (*P* = .01).

## 4. Discussion

This study prospectively evaluated 2005 SPECT appropriateness criteria in a diverse patient and physician population from a large regional medical center. Overall, prevalence of appropriate (63%) and inappropriate (14%) SPECT was similar to those reported by previous studies [11]. The patient population was diverse: nearly equally representative of men and women, patients with or without CAD, inpatients and outpatients, and referred by private as well as full-time faculty cardiologists and noncardiologists. Almost all patients were successfully assigned into an appropriateness category with only 3% not classifiable (Figure 1). Followup was complete in 97.4% of patients, and outcome analysis was undertaken for a combined end point of total mortality, cardiac revascularization, and cardiac admission for reason other than revascularization.

Several important conclusions emerge from this study. First, appropriateness of SPECT was strongly influenced by presence of symptoms (Figure 2(a)), pre-test likelihood of disease (Figure 2(b)), and Framingham risk scores (Figure 3). As may have been expected, there was a strong trend for more appropriate studies in symptomatic patients and those at a greater likelihood of CAD. Importantly, most inappropriate studies were observed in asymptomatic and low risk patients. These findings prove the robustness of criteria in clinical application. While specific scenarios may differ from institution to institution, the recognition of these general patterns in appropriateness criteria should be helpful to clinicians.

Secondly, findings emerged in patient subgroups that are interesting and require further investigation. As may be expected, inpatients were more symptomatic and at a higher clinical probability of coronary artery disease. Thus, it is not surprising that more appropriate SPECT studies were observed in inpatients. Appropriateness criteria appeared more favorable toward patients with established CAD. 

Women were more symptomatic than men but did not differ in pre-test likelihood of disease and had a lower prevalence of known CAD. Among asymptomatic patients, more women were at a lower FRS. While less of appropriate SPECT was observed for women (59% versus 70% in men, Table 3), the most notable difference was in the occurrence of the inappropriate studies (22% in women versus 9% in men). It is likely that such a difference cannot be attributed solely to less prevalent CAD or lower FRS. These findings suggest a gender-related referral bias and indicate that it may be important to include gender-specific recommendations in the appropriateness criteria. 

Another challenging group were asymptomatic patients in an intermediate FRS group (Figure 3). Most of them had uncertain SPECT (74%), some were appropriate (23%), and very few were inappropriate (3%). Performing SPECT in asymptomatic patients may not be warranted, and further studies are needed to determine merits of SPECT imaging in this patient subgroup. The 2009 AUC consider intermediate risk patients with interpretable ECG in the inappropriate, and those with uninterpretable ECG in the uncertain categories. Further discussion of the merits of SPECT MPI and other modalities in the asymptomatic patients is provided in the informational statements on http://asnc.org/imageuploads/Asymptomatic.pdf.

There were notable differences by referring physician specialty. Cardiovascular physicians outperformed their noncardiovascular colleagues with more appropriate SPECT referred, although that difference was small (69% versus 62%). Importantly, significantly fewer inappropriate studies were referred by cardiovascular MDs (10% versus 18%, Table 5). These results may be reflective of a patient selection bias as more “appropriate” patients such as men, and those with known CAD were referred by cardiovascular physicians. However, significantly more inpatients (another “appropriate” group) were referred by noncardiovascular MDs. No significant differences were observed among private and faculty cardiologists. Further educational and consultative efforts are likely needed to increase appropriateness of referrals by noncardiovascular physicians. 

Finally, outcomes differed significantly by appropriateness of SPECT. Unadjusted event rates were high for patients with appropriate SPECT (12%), lower for those with uncertain SPECT (7.1%), and lowest for the inappropriate SPECT patients (2.4%). A 7.1% event rate in the uncertain group suggests that it is prudent to perform these studies until factors or scenarios are identified that would allow to further risk stratify this group.

### 4.1. Limitations

Notable limitations exist in this study. First, this represented a single medical center experience. As such, selection bias, referral bias, and practice preference bias were all present. Second, we have made assumptions in calculating FRS that may have affected appropriateness designations. Third, not all appropriateness indications were equally prevalent in our cohort, and some were observed in less than 1% of the patients. Fourth, we have not been able to evaluate cardiac mortality but rather a combined end point that included total mortality, revascularization, and cardiac admissions. Fifth, 15 of our patients remained unclassified. Most of them would have been assigned as uncertain if 2009 AUC were used, and not likely to affect appropriate and inappropriate groups.

## 5. Conclusion

We have established that appropriateness criteria are effective in identifying appropriateness of SPECT in a diverse patient population. More appropriate studies were observed in patients with symptoms and at higher pre-test likelihood and Framingham risk. Most of the inappropriate studies were observed in asymptomatic and low risk patients. Patients without known CAD, women, and those with an intermediate Framingham risk emerged as subgroups where further investigation is warranted. Physician specialty was an important factor determining appropriateness with cardiovascular physicians outperforming their noncardiovascular colleagues. Unadjusted event rates were highest in the appropriate SPECT group, intermediate in the uncertain group, and lowest for the inappropriate SPECT patients.

## Figures and Tables

**Table 1:** Patient population.

Patient characteristics	*N*; %
Age (mean ± SD)	63.5 ± 13.1 yrs
Admission status	
*Inpatient *	268; 48%
*Outpatient *	317; 54%
Gender	
*Men *	307; 55%
*Women *	266; 45%
Known CAD	
*Yes *	166; 28%
*No *	419; 72%
Symptoms	
*Yes *	307; 53%
* No *	278; 47%
Pretest likelihood	
* Low *	299; 51%
* Intermediate *	253; 43%
* High *	33; 6%
Framingham risk	
* Low *	58; 33%
* Intermediate *	101; 58%
* High *	16; 9%
Test type	
*Exercise *	335; 57%
*Pharmacologic *	249; 43%

**Table 2:** SPECT MPI indications observed in the study: comparison of 2005 and 2009 AUC.

Indications	2005	2009
*Detection of CAD:*		
(i) symptomatic, intermediate or high pretest probability,	A	A
(ii) asymptomatic, moderate Framingham Risk Score (FRS)*,	U	ECG interpret: I, not: U
(iii) asymptomatic, high FRS,	A	A
(iv) asymptomatic, low FRS or symptomatic, ECG interpretable and able to exercise,	I	I

*After revascularization (PCI or CABG):*		
(i) symptomatic,	A	A
(ii) asymptomatic or symptomatic prior to CABG, ≥5 yrs after^†^,	A	A
(iii) asymptomatic or symptomatic prior to PCI, ≥2 yrs after.	U	U

*Prior test results:*		
(i) asymptomatic or stable symptoms, abnormal catheterization or prior SPECT ≥2 yrs to evaluate worsening disease	A	U

*Preoperative evaluation prior to intermediate-high risk noncardiac surgery:*		
(i) clinical risk factors and poor exercise tolerance (<4 METs),	A	A
(ii) no or minor risk factors, normal exercise tolerance (≥4 METs).	I	I

*Unclassified indications:*		
(i) syncope,	—	Low risk: I, int-high: A
(ii) intermediate Duke treadmill score, low FRS,	—	—
(iii) new onset atrial fibrillation, moderate FRS,	—	U
(iv) asymptomatic, prior myocardial infarct of unknown age,	—	U
(v) symptoms unknown before PCI < 2 yrs ago,	—	—
(vi) known CAD; failed PCI,	—	U
(vii) normal SPECT MPI >2 yrs ago, moderate FRS.	—	U

*ATP III in 2009 AUC. ^†^No symptoms prior to revascularization in 2009 AUC.

**Table 3:** SPECT appropriateness by admission status, gender and known CAD.

Patient Variables	Appropriate	Uncertain	Inappropriate	*P* Value
	*N*, %	*N*, %	*N*, %	
*Admission*				
Inpatient (*n* = 256)	184; 72%	33; 13%	39; 15%	.0004
Outpatient (*n* = 305)	179; 59%	83; 27%	43; 14%	
*Gender*				
Men (*n* = 307)	214; 70%	66; 21%	27; 9%	<.0001
Women (*n* = 263)	156; 59%	50; 19%	57; 22%	
*Known CAD*				
Yes (*n* = 158)	117; 74%	34; 22%	7; 4%	<.0001
No (*n* = 412)	253; 61%	82; 20%	77; 19%	

**Table 4:** Differences by gender, admission status, and known history of CAD by specialty of a referring physician.

Patient Variables	CV MD	NON- CV MD	*P* Value
	*N*, %	*N*, %	
*Admission*			
Inpatient (*n* = 265)	90; 34%	175; 66%	<.0001
Outpatient (*n* = 305)	159; 52%	146; 48%	
*Gender*			
Men (*n* = 307)	159; 52%	148; 48%	<.0001
Women (*n* = 263)	90; 34%	173; 66%	
*Known CAD*			
Yes (n=158)	113; 72%	45; 28%	<.0001
No (n=412)	136; 33%	276; 67%	

CV:cardiovascular.

**Table 5:** SPECT appropriateness by physician specialty and practice type. Differences were significant for non-CV and CV MDs (*P* = .03) but not for full-time versus private CV MDs.

Physicians	Appropriate	Uncertain	Inappropriate
	*N*, %	*N*, %	*N*, %
*Non-CV *(*n* = 321)	198; 62%	65; 20%	58; 18%
*CV *(*n* = 249)	172; 69%	51; 21%	26; 10%
*Private *(*n* = 132)	98; 74%	21; 16%	13; 10%
*Full-time faculty *(*n* = 117)	74; 63%	30; 26%	13; 11%

CV: cardiovascular.
